# Amphiphilic Molecular Brushes with Regular Polydimethylsiloxane Backbone and Poly-2-isopropyl-2-oxazoline Side Chains. 2. Self-Organization in Aqueous Solutions on Heating

**DOI:** 10.3390/polym13010031

**Published:** 2020-12-23

**Authors:** Serafim Rodchenko, Alina Amirova, Mikhail Kurlykin, Andrey Tenkovtsev, Sergey Milenin, Alexander Filippov

**Affiliations:** 1 Institute of Macromolecular Compounds of the Russian Academy of Sciences, Bolshoy pr., 31, 199004 Saint Petersburg, Russia; aliram.new@gmail.com (A.A.); mike_x@mail.ru (M.K.); tenkovtsev@yandex.ru (A.T.); afil@imc.macro.ru (A.F.); 2 Enikolopov Institute of Synthetic Polymeric Materials of the Russian Academy of Sciences, Profsoyuznaya, 70, 117393 Moscow, Russia; cephe@mail.ru

**Keywords:** molecular brushes, poly-2-isopropyl-2-oxazoline, polydimethylsiloxane, thermosensitivity, light scattering, turbidimetry, compaction, aggregation, kinetics of self-organization, LCST

## Abstract

The behavior of amphiphilic molecular brushes in aqueous solutions on heating was studied by light scattering and turbidimetry. The main chain of the graft copolymers was polydimethylsiloxane, and the side chains were thermosensitive poly-2-isopropyl-2-oxazoline. The studied samples differed in the length of the grafted chains (polymerization degrees were 14 and 30) and, accordingly, in the molar fraction of the hydrophobic backbone. The grafting density of both samples was 0.6. At low temperatures, macromolecules and aggregates, which formed due to the interaction of main chains, were observed in solutions. At moderate temperatures, heating solutions of the sample with short side chains led to aggregation due to dehydration of poly-2-isopropyl-2-oxazoline and the formation of intermolecular hydrogen bonds. In the case of the brush with long grafted chains, dehydration caused the formation of intramolecular hydrogen bonds and the compaction of molecules and aggregates. The lower critical solution temperature for solutions of the sample with long side chains was higher than LCST for the sample with short side chains. It was shown that the molar fraction of the hydrophobic component and the intramolecular density are the important factors determining the LCST behavior of amphiphilic molecular brushes in aqueous solutions.

## 1. Introduction

At present, stimuli-responsive polymers are increasingly attracting the attention of researchers, due to the wide possibilities of their practical use in such fields as biotechnology, bioengineering, and medicine [1,2,3,4,5,6,7]. An important place among such systems is occupied by stimuli-sensitive polymers with complex architecture, including amphiphilic cylindrical molecular brushes, in the macromolecules of which a directed variation in the number of branch points, the ratio of components, the number and distribution of functional groups over the volume makes it possible to regulate the properties of the polymer. The architecture of grafted macromolecules promotes the formation of unimolecular micelles in their solutions, which are more stable than aggregates formed in solutions of linear polymers [8,9,10,11].

A large number of stimuli-responsive linear polymers of various chemical structures have been synthesized in recent years [12,13,14,15,16,17,18,19,20,21,22]. The synthesis conditions, which make it possible to obtain polymers with a given structure and molar mass characteristics, have been determined and the effect of the structure and molar mass on the physicochemical properties has been established [23,24,25,26,27]. It has been shown that copolymerization is a convenient mechanism for regulating the properties of stimuli-responsive polymers, which allows changing the hydrophilic–hydrophobic balance of macromolecules directionally [28,29,30]. For example, for gradient copolymers of 2-ethyl- and 2-isopropyl-2-oxazoline, linear growth in lower critical solution temperature (LCST) was observed with an increase in the molar fraction of 2-ethyl-2-oxazoline [31]. The ability of the stimuli-responsive polymers to make complex formations with low molecular weight compounds was studied upon variation of the structure of the latter, as well as external conditions (temperature, concentration, medium acidity, etc.) [32,33].

One of the important representatives of stimuli-responsive polymers is thermosensitive polymers, LCST of which is close to the human body temperature. These polymers include some representatives of poly-2-alkyl-2-oxazolines (PAlOx), the use of which in medical applications is due to their biodegradability, biocompatibility, and low toxicity. At present, PAlOx are used in chromatography, bioseparation, heterogeneous catalysis, as well as in the preparation of complexes and conjugates [5,8,34,35]. One of the interesting representatives of PAlOx is poly-2-isopropyl-2-oxazoline (PiPrOx), which is a structural analog of both poly-*N*-isopropylacryamide and polypeptides [36,37,38]. Note that PAlOx are widely used to obtain polymers of complex architecture, which expands the possibilities of controlling the properties of polymeric materials [14,21,39,40,41,42,43]. The field of application of grafted copolymers containing PiPrOx side chains can be expanded by using polydimethylsiloxane (PDMS) as a hydrophobic backbone. PDMS has good prospects for creating amphiphilic macromolecules [43,44]. Siloxane polymers have many unique properties, such as thermal and oxidation stability and flexibility over a wide temperature range, and are physiologically and biologically inert [45].

In the literature, there is extensive material concerning the development of new synthesis ways of graft copolymers, their experimental study, and theoretical description [46,47,48,49,50,51]. In particular, significant progress has been made in the study of molecular brushes constructed from components, which differ greatly in chemical nature [52,53,54,55,56]. One of the demanded classes of such amphiphilic polymers is graft copolymers containing stimuli-sensitive chains. Their behavior in solutions is determined by both structural parameters and external influences. For molecular brushes with poly-2-alkyl-2-oxazoline side chains and polymethacrylate main chain, the effect of the composition on the ability of a copolymer to form unimers or aggregates in water, methanol, and ethanol was revealed [57]. The compaction of macromolecules on heating was recorded in a solution of copolymer with PiPrOx chains grafted to the acrylamide backbone [58]. For the molecular brush with a polyester main chain and side chains of poly-2-ethyl-2-oxazoline, in addition to thermosensitivity, pH-sensitivity was also recorded [9], the mechanism of which is probably similar to that for linear poly-2-alkyl-2-oxazolines [59,60]. The study of these molecular brushes in aqueous solutions showed that the nature of self-organization depends on the grafting density *z* of side chains [61]. Using the molecular brushes with poly-N-isopropylacrylamide side chains, the influence of the structure of terminal groups on the behavior of aqueous solutions with varying temperature was analyzed [62]. Despite significant advances, many features of the solution behavior of stimulus-responsive graft copolymers remain unclear. Among such problems are the following: the effect of molar mass on LCST behavior of molecular brushes, dependence of their properties on hydrophilic-hydrophobic balance, kinetics of self-organization of molecules in solutions, etc.

Our previous work described the synthesis, characterization, and conformation in a solution of amphiphilic molecular brushes PDMS-*g*-PiPrOx with a regular polydimethylsiloxane backbone and poly-2-isopropyl-2-oxazoline side chains [49]. The amphiphilic structure of molecular brush PDMS-*g*-PiPrOx determines the selectivity of most solvents for them. The PDMS-*g*-PiPrOx molecules in a solution are similar to star-shaped molecules with a collapsed backbone as a core and the PiPrOx chains as arms.

The aim of the present work is to investigate the effect of the length of grafted PiPrOx chains and, accordingly, the hydrophobicity of the molecules on self-organization and aggregation in water solutions of thermoresponsive graft copolymers PDMS-*g*-PiPrOx on heating. To achieve this purpose, the molecular brush samples with different length of PiPrOx chains but with the same length of PDMS backbone and grafting density of the side chains were investigated.

## 2. Materials and Methods 

### 2.1. Characteristics of Investigated Samples of PDMS-g-PiPrOx 

**Scheme 1:**
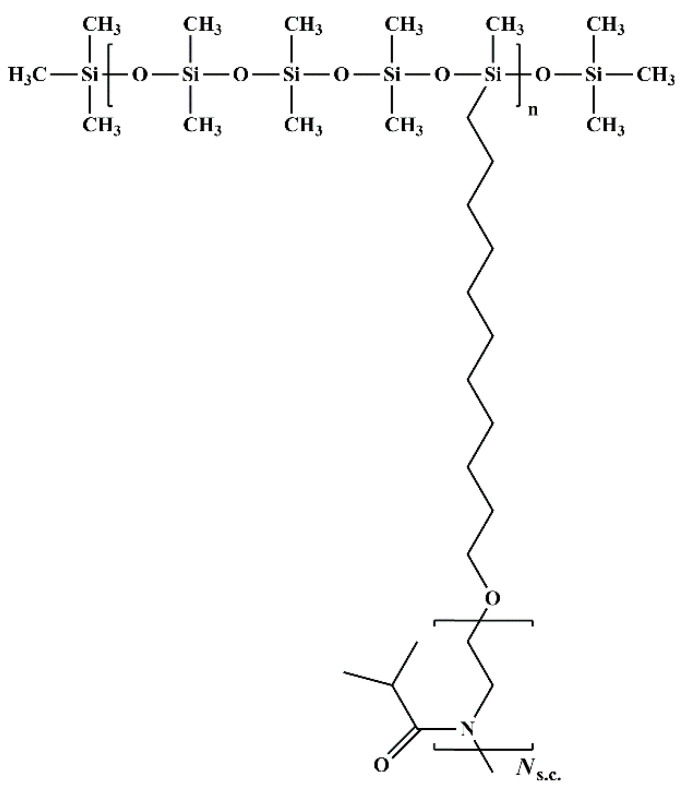
Structure of repeating unit of grafted copolymers polydimethylsiloxane (PDMS)-*g*-poly-2-isopropyl-2-oxazoline (PiPrOx).

The synthesis and characterization of grafted copolymers PDMS-*g*-PiPrOx were described in detail previously [49]. The structure of the repeating unit of PDMS-*g*-PiPrOx is shown in Scheme 1. Regular polydimethylsiloxane with undecene tosylate groups in every fourth siloxane unit was used as a macroinitiator. Its average molar mass number was 65,000 G·mol^−1^. The molecular brush samples with PiPrOx side chains were synthesized by the “grafting from” method. The grafting density of the side chain was equal to *z* = 0.6, i.e., one PiPrOx side chain was accounted for approximately every seventh siloxane group of backbone.

In the present work, two samples of PDMS-*g*-PiPrOx differing in the length of the grafted chains were investigated. The molar masses of samples were obtained by the light scattering method in dilute solutions in 2-nitropropane [49]. The molar masses are 400,000 g·mol^−1^ for Samples 1 and 710,000 g·mol^−1^ for Samples 2. Therefore, the polymerization degree *N*_s.c._ of PiPrOx chains is 14 and 30 for Sample 1 and Samples 2, respectively. The difference in the length of the side chains at a constant length of the backbone determines the difference in the hydrophilic–hydrophobic balance of the samples; the mole fraction ω of the hydrophobic main chain is 28.8 mol % for Samples 1 and 16.0 mol % for Samples 2.

### 2.2. Investigation of Self-Organization in Aqueous Solutions

The thermoresponsive behavior of PDMS-*g*-PiPrOx was investigated in aqueous solutions by method of static (SLS) and dynamic (DLS) light scattering and turbidimetry. A bidistilled water (viscosity η_0_ = 0.981 cP, density *ρ*_0_ = 0.998 g·cm^−3^, and refractive index *n*_0_ = 1.333) was used as a solvent. Solutions were studied in the concentration range from 0.0002 to 0.0050 g·cm^−3^ for both samples. All solutions were filtered into dust-free vials using Millipore filters with the hydrophilic PTFE membrane and the pore size of 0.45 μm (Merck, Darmstadt, Germany).

**Figure 1:**
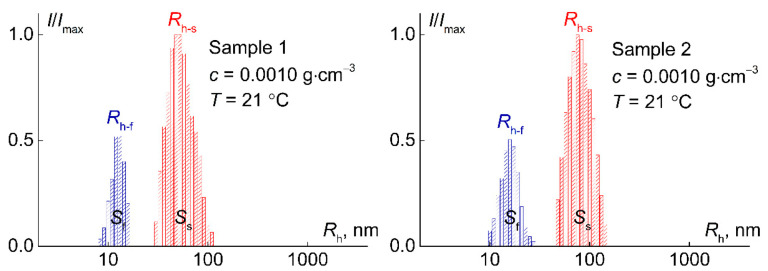
The dependences of relative intensity *I*/*I*_max_ of scattered light on the hydrodynamic radius *R*_h_ of scattering species for PDMS-*g*-PiPrOx solutions. *I*_max_ is the maximum value of light scattering intensity *I* for a given solution.

The experiments were carried out on the Photocor Complex instrument (Photocor Instruments Inc., Moscow, Russia) equipped with a Spectra-Physics He-Ne laser (wavelength λ_0_ = 659.1 nm), a Photocor-FC2 correlator with 288 channels, and a Photocor-PD detection device for measuring the intensity of the transmitted light. The solution temperature *T* was changed discretely over the interval from 15 to 75 °C with the steps ranging from 1.0 °C (near cloud point) to 5 °C (at low temperatures). The temperature was controlled with an accuracy of 0.1 °C. The autocorrelation function was processed with a DynaLS soft (ver. 8.2.3, SoftScientific, Tirat Carmel, Israel). The data were obtained by regularization method using a singular value decomposition, based on a multi-pass algorithm, which is more stable for separating the resulting peaks in the presence of a large amount of noise in the received data. Toluene was used as a calibration liquid. Hydrodynamic radii *R*_h_ of the scattering objects were obtained by the DLS method analyzing the autocorrelation function. The contributions *S*_i_ of ith type of species to the integral scattering intensity *I* measured as squares under the curved line of the intensity distribution corresponding peak on *R_h_* (Figure 1).

**Figure 2:**
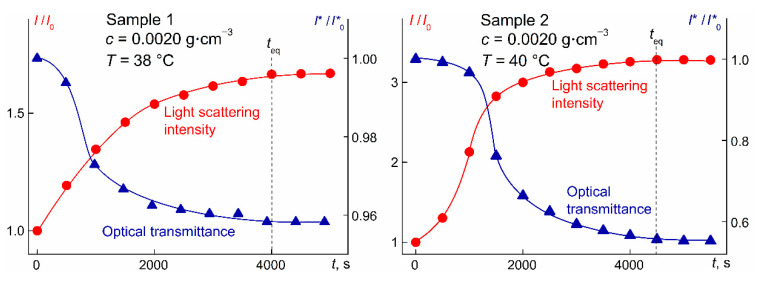
Time dependencies of intensities of light scattering *I* and transmitted light I* for the aqueous solution of PDMS-*g*-PiPrOx. *I*_0_ and I*_0_ are intensities of light scattering and optical transmittance at *t* = 0, respectively.

The procedure was as follows: after reaching the given temperature, the analysis of light scattering intensity *I* and optical transmittance I* started. At each temperature, we assumed that *t* = 0 was the moment when the sample reached the required temperature. In these conditions, the experiments were carried out at a 90° scattering angle. Figure 2 shows the time dependences of *I* and I*. The time *t*_eq_, during which the light scattering intensity and the optical transmission reach constant time values, was used as a characteristic of the duration of establishing of the “equilibrium” system state. The accuracy of determining the *t*_eq_ value was about 5–10%, depending on the relative change in *I* and I*.

**Figure 3:**
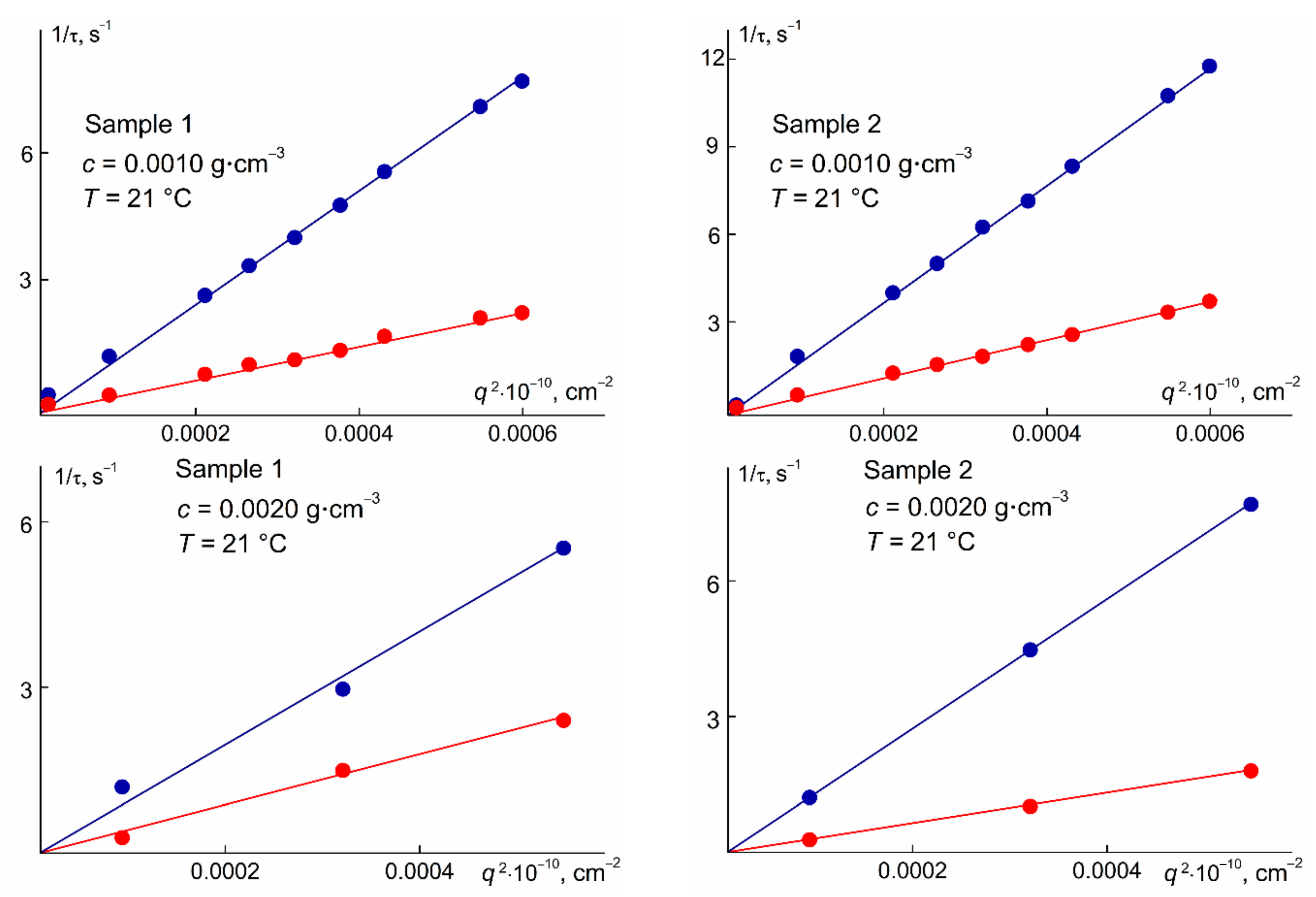
The dependences of the inverse relaxation time 1/τ on the squared wave vector *q*^2^ for fast (blue symbols) and slow modes (red symbols) for solutions of PDMS-*g*-PiPrOx.

The hydrodynamic radii *R*_h_ of dissolved particles and their contributions *S*_i_ to the integral scattering intensity *I* were measured when *I* became time independent. To confirm the diffusion processes, the values of *I*, *R_h_* and *S*_i_ were analyzed in the range of scattering angles from 30 to 140 °. The correctness of the estimation of the hydrodynamic radii can be verified by checking the linearity of the dependence of the correlation rate 1/τ (τ is the relaxation time) on *q*^2^ (*q* is the wave vector). Figure 3 shows the presence of two modes for solutions under study and confirms the diffusion character of these modes because the dependences, treated by the cumulant method for solutions of both samples, are linear and pass through the origin.

## 3. Results

### 3.1. Behavior of Molecular Brush Solutions at Low Temperatures

**Figure 4:**
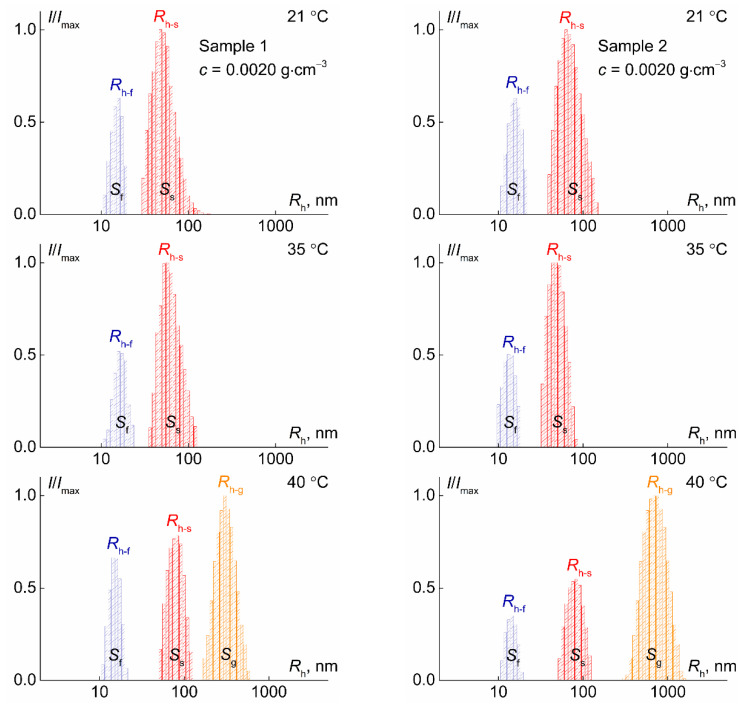
The dependences of relative intensity *I*/*I*_max_ of scattered light on the hydrodynamic radius *R*_h_ of scattering species for PDMS-*g*-PiPrOx solutions.

**Figure 5:**
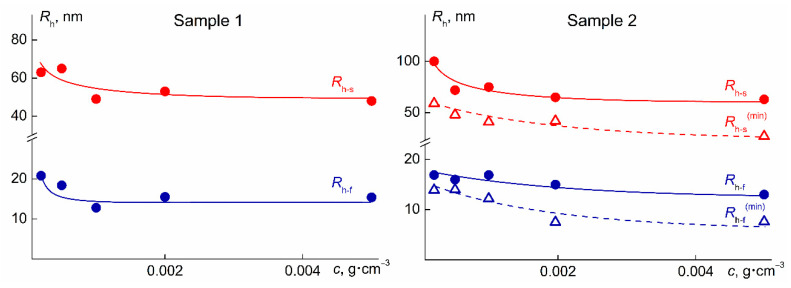
The dependences of the hydrodynamic radius *R*_h_ of scattering species on concentration *c* for aqueous solutions of investigated molecular brushes at 21 °C.

At low temperatures, for solutions of both studied samples, the distribution of scattered light intensity over hydrodynamic radii was bimodal (Figure 4). Consequently, there were two types of scattering objects with hydrodynamic radii *R*_h-f_ (particles responsible for the fast mode) and *R*_h-s_ (particles responsible for the slow mode). The existence of two or even more types of species in solutions of stimulus-sensitive molecular brushes was observed quite often [8,9,10,11,63,64,65], and the size and fraction of various particles depended on the grafting density of side chains, their length, and hydrophilic–hydrophobic balance, as well as on the polymer concentration and the medium acidity. In the case of the studied solutions, the radii *R*_h-f_ and *R*_h-s_ increased with dilution (Figure 5), which probably reflects the concentration dependence of the translational friction coefficient *f* [66,67,68,69].

At relatively high concentrations (*c* > 0.001 g·cm^−3^), the *R*_h-f_ radii within the experimental error coincide with the values of the hydrodynamic radii of macromolecules *R*_h-f_ = 14 and 13 nm for Samples 1 and 2, respectively, which were determined in solvents in which associative phenomena were not observed [49]. Therefore, we can conclude that the particles responsible for the fast mode are individual molecules of the studied graft copolymers. In addition, it can be assumed that in the aqueous solutions, their macromolecules have the same conformation as in organic selective solvents, namely, they resemble the molecules of polymer star, the core of which is a strongly folded PDMS main chain, and the arms are grafted PiPrOx chains [49].

The slow mode describes the diffusion of aggregates. At low temperatures, the aggregate formation is mainly caused by the interaction of the main PDMS chains of macromolecules, for which the grafting density *z* of the side chains is noticeably lower than the average value of *z* for the ensemble of macromolecules, as was previously observed for molecular brushes APE-*g*-PAlOx with an aromatic polyester (APE) main chain and PAlOx side chains [8,9,11]. Another reason for aggregation may also be the interaction of 2-isopropyl-2-oxazoline units, which have lost their solubility in water. Indeed, it has been shown that PiPrOx dehydration can begin at a temperature of about 20 °C [70].

The contribution *S*_s_ of aggregates to the integral value of the light scattering intensity for both samples significantly exceeded the corresponding *S*_f_ characteristic for macromolecules. No dependence of *S*_s_ and *S*_s_ on concentration was found, and the average values of *S*_s_/*S*_f_ were 4 ± 1 for Sample 1 and 3 ± 1 for Sample 2. Using the values of *S*_s_ and *S*_s_, one can estimate the relative weight fractions of molecules *c*′_f_ and aggregates *c*′_s_ in solutions. According to the static light scattering theory [71,72,73], the intensity *I*_i_ of *i*th species is proportional to both the molar mass *M*_i_ and concentration *c* of particles as follows from the simplified form of the Rayleigh equation

*I*_i_ ~ *c*_i_*M*_i_ (1)

The particle radius *R*_i_ is related to its molar mass as *M*_i_ ~ *R*_i_*^x^*, where parameter *x* depends strongly on the particle shape. To calculate the values of the ratio *c*′_s_/*c*′_f_, one can use the models of spherical particles (*x* = 3) for macromolecules and a loose coil (*x* = 2) for aggregates. Within this approximation, we obtain

*c*′_s_/*c*′_f_ = (*S*_s_/*R*_h-s_^2^)/(*S*_f_/*R*_h-f_^3^) (2)

Thus, rough estimation shows that for both samples *c*′_s_/*c*′_f_ ≈ 3 ± 1, therefore the relative weight fraction of the aggregates *c*′_f_ at low temperatures exceed 70%.

To estimate roughly the aggregation degree *m*_a_ from the values of the hydrodynamic radii *R*_h-f_ and *R*_h-s_, the models of the rigid sphere for macromolecules and an ellipsoid of revolution for aggregates were used. Moreover, it was assumed that the densities of aggregates and molecules were the same, more precisely, that the fractions of a polymer substance in the volume of a macromolecule and an aggregate were the same.

A rigorous solution of the hydrodynamic Navier–Stokes equation [74] leads to the well-known Stokes equation for translational frictional coefficient *f*

*f* = 6πη_0_*R*_h-D_ (3)

where η_0_ is the liquid viscosity. For an aspherical body, for example, the ellipsoid of revolution of the translational frictional coefficient is a tensor value. Therefore, the *f* value depends on the direction of motion with respect to a system of coordinates related to the particle. If the orientation distribution in an assemble of an elongated ellipsoid of revolution is random, the average value of the translational frictional coefficient is given by (see, for example, [75])

*f* = 6πη_0_·*a*·(*p*^2^ − 1)^1/2^/(*p*·ln((*p* + (*p*^2^ − 1)^1/2^)/(*p* − (*p*^2^ − 1)^1/2^)) = 6πη_0_·*aF*(*p*) (4)

where *p* = *a*/*b*, *a* and *b* are the major and minor axes of ellipsoid. Substituting the *f* value into Equation (4), it is easy to obtain the dependences of *a* and *b* on *p* for ellipsoids of revolution with a given friction coefficient *f.*

*a* = *f*/(6πη_0_*F*(*p*)) (5)

*b* = *f*/(6πη_0_*pF*(*p*)) (6)

In accordance with the made approximation, the aggregation degree *m*_a_ is equal to the ratio of the volumes of the aggregate and the macromolecule. The volume of the macromolecule can be expressed as the volume of the modeling sphere *V*_sph_ = 4π/3·*R*_h-f_^3^, and the volume of the aggregate is the volume of the modeling ellipsoid of revolution *V*_ell_ = 4π*ab*^2^/24 = 4π*a*^3^/24*p*^2^. Accordingly, the aggregation degree *m*_a_ can be represented as a function of the asymmetry factor *p*

*m*_a_ = *V*_ell_/*V*_sph_ = *p*^2^*R*_h-f_^3^/8*a*^3^ (7)

**Figure 6:**
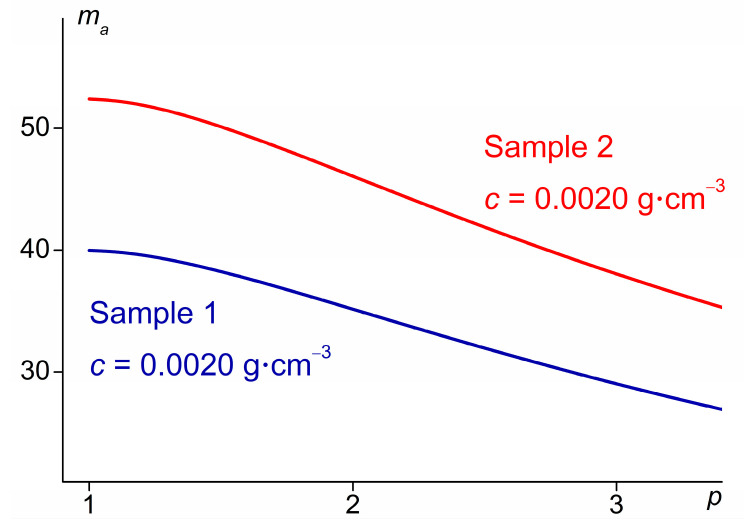
The dependences of aggregation degree *m*_a_ on asymmetry factor *p* of modeling ellipsoid for aqueous solutions of PDMS-*g*-PiPrOx.

Figure 6 shows the dependences (7) for a solution of Sample 1 and 2 with a concentration of *c* = 0.0020 g·cm^−3^. Similar dependences were obtained for all studied solutions. It is clearly seen that in the region of “reasonable” values of the asymmetry factor *p* < 3, i.e., *p*, which can be expected for aggregates, the aggregation degree does not change strongly. The change in *m*_a_ is about 30% with increasing *p* from 1 to 3. For both samples, at a given *p* value, no systematic change in *m*_a_ with concentration was detected. At *p* = 2, the average values of *m*_a_ are 35 ± 5 and 80 ± 15 for Samples 1 and 2, respectively. Consequently, at room temperature, the aggregates contain several tens of macromolecules. Note again that the calculated values of *m*_a_ are rough estimates. A more reliable way to determine the aggregation degree is to compare the molar masses of the polymer *M*_m_ and aggregates *M*_a_, namely, *m*_a_ = *M*_a_/*M*_m_. However, due to the large experimental error in determining the contribution of aggregates *S*_s_ to the total light scattering intensity for the studied solutions, it was not possible to obtain a reliable angular dependence of the inverse light scattering intensity. We can only speak about the order of magnitude of *M*_a_ and, accordingly, the degree of aggregation. For both samples, the interval of the experimental values of *m*_a_ is 10–300.

### 3.2. Dependence of the Characteristics of PDMS-g-PiPrOx Aqueous Solutions on Temperature

**Figure 7:**
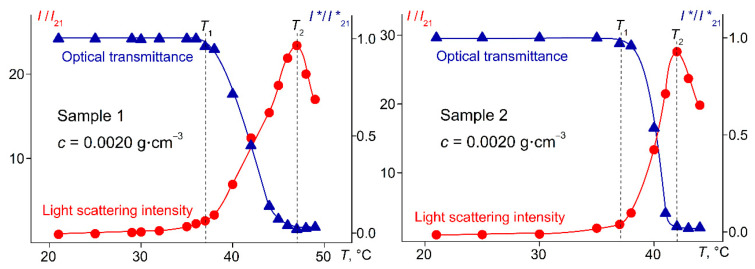
Temperature dependences of relative light scattering intensity *I*/*I*_21_ and relative transmission I*/I*_21_ for solutions of investigated grafted copolymers. *I*_21_ and I*_21_ are light scattering intensity and optical transmission at 21 °C, respectively.

On heating, a structural-phase transition was observed in all the solutions studied. The temperatures of the onset *T*_1_ and finishing *T*_2_ of the phase separation were determined turbidimetrically as the temperatures at which a sharp decrease in the optical transmission I* began and at which this characteristic became zero, respectively (Figure 7). The second method used for determining *T*_1_ and *T*_2_ was an analysis of the temperature dependences of the light scattering intensity *I*. At temperature *T*_1_, a strong increase in *I* was observed; at *T*_2_, the light scattering intensity reached the maximum value and then began to decrease.

**Figure 8:**
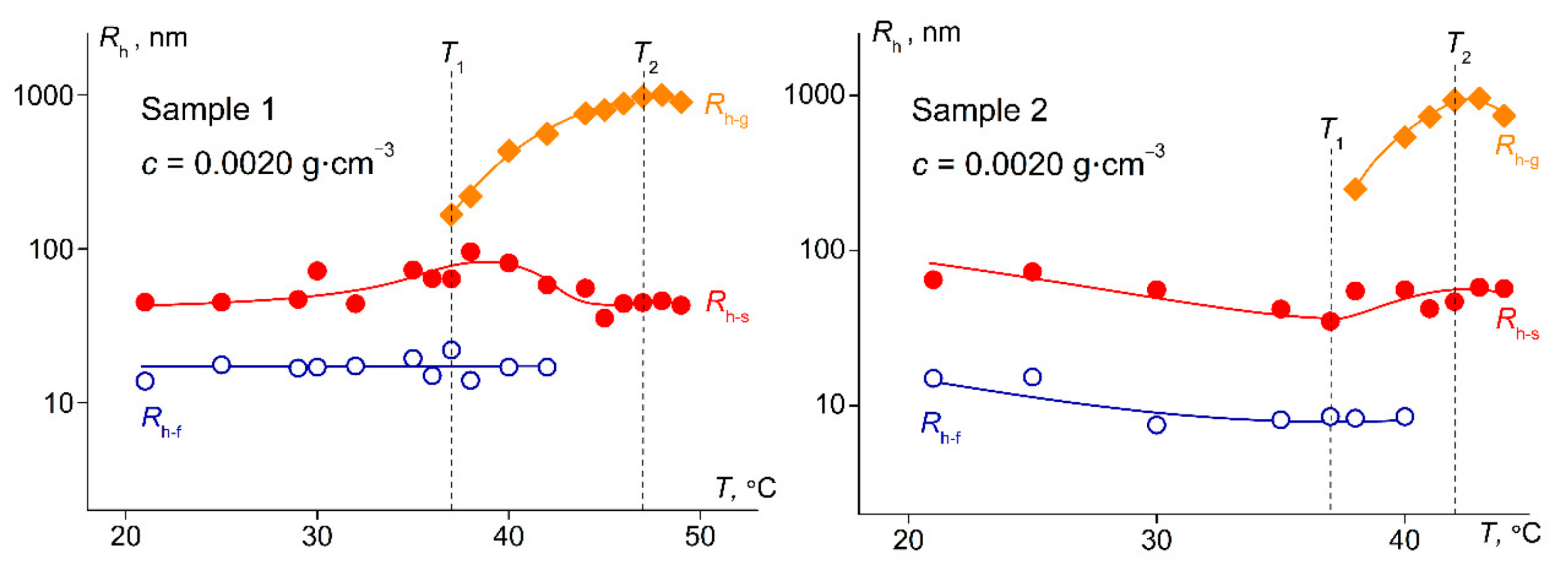
Temperature dependences of hydrodynamic radii *R*_h_ of scattering objects for aqueous solutions of PDMS-*g*-PiPrOx.

At *T* < *T*_1_, a slow increase in the light scattering intensity with temperature was observed for solutions of both samples; however, the processes occurring in solutions of Samples 1 and 2 were different. As can be seen in Figure 8, in the considered temperature range, the hydrodynamic dimension of the aggregates increased on heating. Consequently, at *T* < *T*_1_, the dominant process in solutions of Sample 1 was the macromolecule aggregation, which was caused by an increase in the average degree of dehydration of PiPrOx with temperature growth and the formation of intermolecular hydrogen bonds.

In the case of Sample 2, the dehydration of PiPrOx led mainly to the realization of intramolecular hydrogen bonds and the compaction of molecules, as evidenced by a decrease in the hydrodynamic radii of scattering objects (Figure 4 and Figure 8). The minimum values of the radii of molecules *R*_h-f_^(min)^ and aggregates *R*_h-s_^(min)^ were observed near the temperature *T*_1_ of the onset of phase separation. As well as the *R*_h-f_ and *R*_h-s_ values at room temperature, the values of *R*_h-f_^(min)^ and *R*_h-s_^(min)^ increased with dilution at low concentrations (Figure 5). Note that the relative change in the radii of molecules and aggregates was different. Indeed, for supramolecular structures, the concentration-average value of the ratio *R*_h-s_^(21)^/*R*_h-s_^(min)^ = 1.7, and for molecules, a similar ratio *R*_h-f_^(21)^/*R*_h-f_^(min)^ = 1.4 (*R*_h-f_^(21)^ and *R*_h-s_^(21)^ are the values of the hydrodynamic radii of macromolecules and aggregates at 21 °C, respectively). This discrepancy can be explained by the different densities of aggregates and macromolecules. The density of the particles under consideration is the fraction of the polymer substance per unit of their volume. For steric reasons, dense macromolecules compact less than aggregates. On the other hand, the difference between the *R*_h-s_^(21)^/*R*_h-s_^(min)^ and *R*_h-f_^(21)^/*R*_h-f_^(min)^ values is small. This fact indicates indirectly the close densities of polymer molecules and aggregates built from them. Qualitatively similar phenomena were previously observed for solutions of graft copolymers APE-*g*-PAlOx [8,9,11] and star-shaped PAlOx [76,77,78]. However, in the latter case, a difference in the compaction degree was found not for molecules and aggregates, but for different supramolecular structures, namely, for micelle-like aggregates and large loose aggregates.

**Figure 9:**
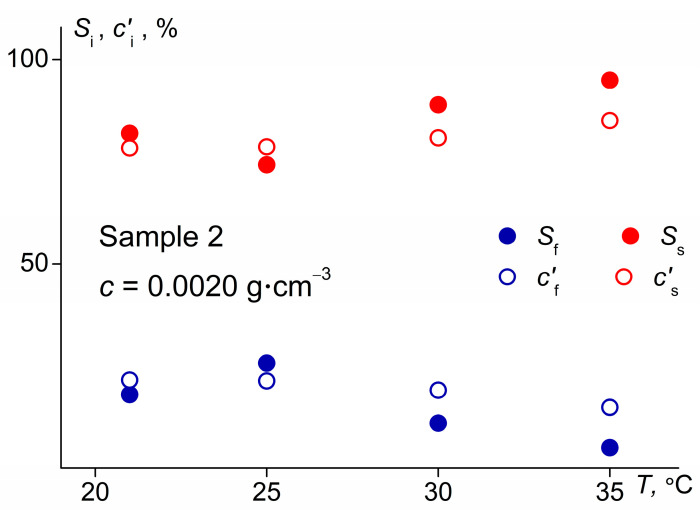
The dependences of S*_f_, S*_s_, *c*′_f_ and *c*′_s_ on temperature for aqueous solution of Sample 2 with concentration *c* = 0.0020 g·cm^−3^.

At *T* < *T*_1_, despite a decrease in the size of the scattering objects (Figure 8), the light scattering intensity by solutions of Sample 2, as indicated above, increased on heating (Figure 7). This behavior was caused by a change in the ratio of aggregates and macromolecules in solutions. As can be seen in Figure 9, with growth of temperature, the contribution of aggregates *S*_s_ to the integrated light scattering intensity increased, while the contribution of molecules *S*_f_ decreased, which reflected an increase in the relative concentration of aggregates *c*′_s_ on account of a decrease in the fraction of molecules *c*′_f_. The *c*′_s_ and *c*′_f_ values shown in Figure 9 were obtained from Equation (2) using models of spherical particle for polymer molecules and an ellipsoid of revolution for supramolecular structures.

Thus, at *T* < *T*_1_, two processes occur in aqueous solutions of PDMS-*g*-PiPrOx, the aggregation of molecules and their compaction. However, in the case of a brush with shorter grafted chains, aggregation dominates, while in solutions of Sample 2, the compaction of macromolecules and aggregates is more pronounced. These facts can be explained by the difference in the molar fraction ω of the hydrophobic main PDMS chain and in the intramolecular density of the studied samples. An increase in ω (Sample 1) facilitates the contacts of the main chains, favoring the aggregation processes. Elongation of the PiPrOx side chains (Sample 2) leads to an increase in the size and density of the hydrophilic shell, which shields the main chain from the solvent. This reduces the probability of contacts between the main chains of different macromolecules and, therefore, prevents aggregation. A similar effect is caused by a change in the grafting density *z* of side chains at a given length, which was observed, for example, for APE-*g*-PAlOx [8]. As well as shortening the grafted chains, a decrease in *z* causes an increase in fraction ω of the hydrophobic backbone and a reduction of intramolecular density.

Within the interval from *T*_1_ to *T*_2_, aggregation processes prevailed in aqueous solutions of both samples. New “giant” supramolecular structures were formed, the hydrodynamic radii *R*_h-g_ of which increased with temperature growth, reaching a maximum value (one micron and higher) near the temperature *T*_2_ (Figure 4 and Figure 8). The contribution of these particles to the total light scattering intensity also increased with temperature. In the considered temperature range, the *R*_h-s_ radii were weakly dependent on temperature, and macromolecules were not detected by the dynamic light scattering method (Figure 8). Thus, at *T*_1_ < *T* < *T*_2_, the particles that presented in solutions at low temperatures were combined into new “giant” particles. For most of the investigated solutions, near the temperature *T*_2_, the *I* values and the aggregates size reached maximum values. Above *T*_2_, the light scattering intensity and the aggregate size began to decrease (Figure 7 and Figure 8), which may be caused by the compaction of molecules and, accordingly, aggregates. Therefore, compaction prevailed within the discussed temperature interval. However, a quantitative interpretation of the results at *T* > *T*_2_ is impossible, since the solutions are cloudy and light scattering is not classical.

**Figure 10:**
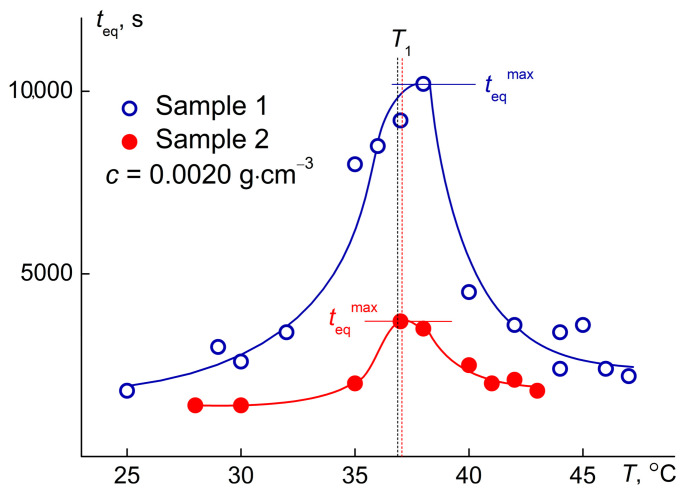
Temperature dependence of *t*_eq_ for solutions of investigated samples.

The length of the grafted chains also influenced the rate of the processes of establishing time-constant characteristics of the solutions after a jump-like change in temperature *T*. Figure 10 compares the temperature dependences of the time *t*_eq_ obtained for aqueous solutions of Samples 1 and 2 with the same concentration. Similar dependences were obtained for all studied solutions. The character of changes of *t*_eq_ with temperature is similar to that observed for star-shaped PAlOx and APE-*g*-PAlOx graft copolymers [8,11,61,79]. At moderate temperatures, the *t*_eq_ times increased on heating. The maximum values of the “establishment” time *t*_eq_^(max)^ were observed in the vicinity of *T*_1_. With further growth of temperature, *t*_eq_ decreased to the values obtained at low temperatures. Figure 10 clearly shows that at all temperatures the duration of the “establishment” processes for Sample 1 is higher than for Sample 2. It can be assumed that the value of *t*_eq_ depends on the character of self-organization processes in the solutions of the studied samples. It is clear from general considerations that compaction is a faster process than aggregation, so the *t*_eq_ value for Sample 2 is lower. However, it should be noted that, for the APE-*g*-PAlOx graft copolymers, no influence of the grafting density on the duration of the processes of establishing equilibrium values of characteristics was found [8].

For both samples of PDMS-*g*-PiPrOx at low temperatures, *t*_eq_ was in the range from 1500 to 3000 s. The same values of *t*_eq_ were obtained at *T* → *T*_2_. The maximum “settling” times *t*_eq_^(max)^ exceeded 10,000 s for Sample 1 and 6000 s for Sample 2. The *t*_eq_ and *t*_eq_^(max)^ values obtained for the investigated molecular brushes are greater than the typical “settling” times for linear thermosensitive polymers [26,80,81]. For other molecular brushes, the *t*_eq_^(max)^ values of similar order of magnitude were obtained: for APE-*g*-PAlOx, the time *t*_eq_^(max)^ reached 11,000 s [31,63], for grafted copolymer of chitosan and poly(N, N-diethylacrylamide) (CS-*g*-PDEAA) it did not exceed 9000 s [10], and for copolymer with polyimide backbone and poly(N,N-dimethylaminoethyl methacrylate) side chains (PI-*g*-PDMAEMA) time *t*_eq_^(max)^ ~ 10,000 s [63]. Note that the molar masses *M* of the compared samples differed strongly. CS-*g*-PDEAA had the maximum *M* = 950,000 g·mol^−1^, and for PI-*g*-PDMAEMA the *M* value was two times lower (470,000 g·mol^−1^). The APE-*g*-PAlOx samples were characterized by the lowest molar masses; for them, *M* ranged from 59,000 to 75,000 g·mol^−1^. Therefore, we can conclude that molar mass is not a determining factor affecting the rate of processes occurring in solutions of stimulus-sensitive molecular brushes. This conclusion is confirmed by the data for the star-shaped PiPrOx [61,78,82], the molar mass of the samples of which was about 22,000 g·mol^−1^, and the *t*_eq_^(max)^ values exceeded 40,000 s. The intramolecular density, which increases with the passage from the studied brushes to the eight-arms stars, is probably critical.

### 3.3. Phase Separation Temperatures of PDMS-g-PiPrOx Aqueous Solutions

**Figure 11:**
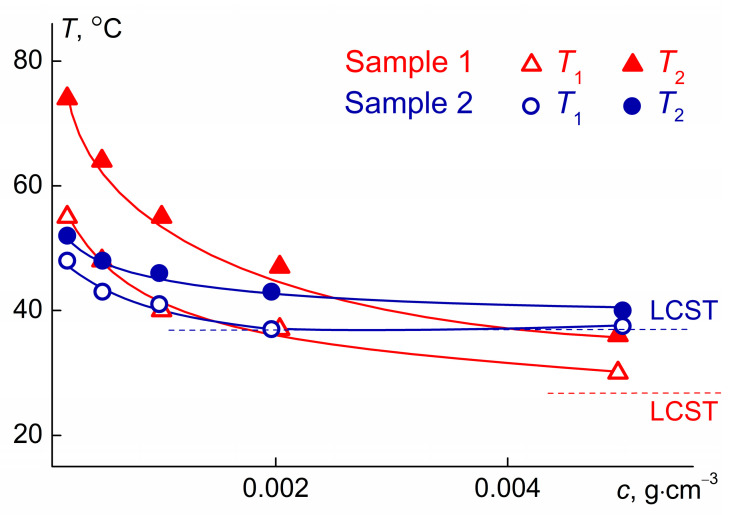
Concentration dependences of the temperatures *T*_1_ and *T*_2_ for solutions of investigated brushes.

Intramolecular density also significantly affects the phase separation temperatures. Figure 11 demonstrates the concentration dependences of the temperatures of the onset *T*_1_ and finishing *T*_2_ of the phase transition. At low concentrations (*c* < 0.001 g·cm^−3^), the temperature boundaries of phase separation increase with dilution. This is typical for solutions of thermosensitive polymers in the diluted region [83,84,85,86,87,88]. For Sample 1, the widening of the phase separation interval *T*_2_ − *T*_1_ is also detected with dilution. At the high concentration, *T*_2_ − *T*_1_ decreased, which probably reflected the semi-dilute regime of the solutions. In the case of Sample 2, *T*_2_ − *T*_1_ is weakly dependent on concentration. Furthermore, in the studied concentration range, the width of the phase separation interval for the brush with short grafted chains is less than *T*_2_ − *T*_1_ for Sample 2.

For both samples, with increasing concentration, the rate of change in *T*_1_ and *T*_2_ decreased. Note that for Sample 1 the width of the phase separation interval (*T*_2_ − *T*_1_) increases with dilution, while for Sample 2 the difference *T*_2_ − *T*_1_ is practically independent of concentration. This difference is a consequence of the different intramolecular density. For the sample with long side chains, a slight increase in *T*_1_ was observed for the most concentrated solution. Therefore, the LCST for solutions of Sample 2 is equal to or slightly less than 37 °C. Taking into account the character of the dependence of *T*_1_ on *c*, it can be expected that LCST for Sample 2 does not differ much from 27 °C. Thus, for the lower molar mass sample, the LCST is about 10 °C lower than for solutions of the high molar mass brush. As is known, for linear thermosensitive polymers, cloud point and LCST decrease usually with increasing molar mass [89,90,91]. Probably, in the case of the studied polymer brushes, the decisive factors regulating the LCST value are the molar fraction of the hydrophobic main chain and the intramolecular density. An increase in the molecular hydrophobicity leads to a decrease in LCST, and a high density of the hydrophilic shell makes it difficult to contact hydrophobic chains and groups and, accordingly, increases LCST.

It is often assumed that the passage from a linear polymer to a polymer with complex architecture is accompanied by a decrease in the phase separation temperatures. In our opinion, the value of cloud point and LCST is influenced by a combination of many factors, such as hydrophilic-hydrophobic balance, molar mass, architecture parameters, conformation of macromolecules, conformation of macromolecular components. The complexity of the problem is evidenced by the data obtained by the authors for linear, star-shaped, and grafted polymers with PiPrOx chains [22,78,82,92]. For example, with an increase in the arm number of star-shaped PiPrOx, the cores of which were carbosilane dendrimers, the phase separation temperatures decreased. These stars were characterized by similar hydrophobic–hydrophilic balance and arm lengths, but differed in the core surface area per arm [22,92]. Solutions of linear analogs of arms had phase separation temperatures above or below the LCST for stars, depending on the hydrophobicity of linear molecules [22,92]. The hydrophobicity of the studied brush with long side chains practically coincided with that for the described eight-arm star, and the molar masses of the star and the brush differed 20 times. Despite such a significant difference in molar masses, the LCSTs for the compared polymers differed by no more than two degrees. We also note that for the eight-arm PiPrOx with the calix [8] arene core [82], the LCST is lower than for the PiPrOx star with a dendrimer core, although the latter has a higher molar mass.

## 4. Conclusions

Self-organization in aqueous solution of amphiphilic molecular brushes with differing lengths of PiPrOx side chains were investigated within a wide concentration and temperature ranges. The grafting density *z* of the side chains of both studied samples was equal to 0.6. At low temperatures, there were two types of scattering objects, namely, macromolecules and aggregates. Conformation of macromolecules resemble a polymer star, the core of which is a strongly folded hydrophobic PDMS backbone and the arms are PiPrOx chains. The aggregates formed as a result of the interaction of PDMS main chains. At room temperature, the aggregates contained several tens of macromolecules.

At temperatures below the phase separation temperature, the processes occurring in aqueous solutions of the studied samples on heating differed significantly. In solutions of the sample with short side chains and high fraction of hydrophobic component, the aggregation prevailed due to the increase in the degree of dehydration of PiPrOx with temperature growth and the formation of intermolecular hydrogen bonds. The elongation of the side chains leads to an increase in the density of the hydrophilic shell, which prevents the contacts between the backbones of different macromolecules. Therefore, for the sample with long grafted chains, a dehydration caused the formation of intramolecular hydrogen bonds and the compaction of molecules and aggregates. The increase in intramolecular density is probably also the reason for the increase in the rate of the processes of establishing time-constant characteristics of the solutions after a jump-like temperature change. Within the phase separation interval, the aggregation dominated in solutions of both samples, and new “giant” supramolecular structures were formed.

The phase separation temperatures of both samples decrease with concentration. The lower critical solution temperature for solutions of the sample with long side chains is higher than LCST for samples with short PDMS chains, despite the fact that the molar mass of the latter is almost two times lower. Hence, the molar mass is not a decisive factor in the thermosensitivity of amphiphilic molecular brushes. The more important factors determining the value of LCST and the nature of self-organization in solutions of such brushes are the molar fraction of the hydrophobic component and the intramolecular density.
